# The CD40–CD154 Costimulatory Axis Confers Broad-Spectrum Antiviral Activity Against VHSV and LMBV via NF-κB-Mediated Immune Activation in Largemouth Bass (*Micropterus salmoides*)

**DOI:** 10.3390/ani16111719

**Published:** 2026-06-04

**Authors:** Wanwan Zhang, Ziling Qin, Huifang Zeng, Meisheng Yi, Kuntong Jia

**Affiliations:** 1 School of Marine Sciences, Sun Yat-sen University, Guangzhou 510275, China; zhangww68@mail.sysu.edu.cn (W.Z.); qinzling@mail2.sysu.edu.cn (Z.Q.); zenghf6@mail2.sysu.edu.cn (H.Z.); 2 Guangdong Provincial Key Laboratory of Marine Resources and Coastal Engineering, Guangzhou 510006, China; 3 Southern Marine Science and Engineering Guangdong Laboratory (Zhuhai), Zhuhai 510275, China

**Keywords:** *Micropterus salmoides*, CD40–CD154 costimulatory axis, VHSV, LMBV, NF-κB signaling

## Abstract

The CD40–CD154 receptor-ligand axis is a core costimulatory regulator of antiviral immunity in mammals, but its functional role in teleosts remains largely unknown. Here, we identified the CD40 and CD154 homologs (MsCD40 and MsCD154) from largemouth bass (*Micropterus salmoides*), a globally farmed perciform teleost. Bioinformatic analysis confirmed that MsCD40 and MsCD154 harbor the conserved domain architectures of tumor necrosis factor receptor superfamily and TNF superfamily, respectively, with a teleost-specific phylogenetic clustering pattern. Both genes were ubiquitously expressed in immune-relevant tissues, and their transcription was dynamically regulated in response to viral hemorrhagic septicemia virus (VHSV) and largemouth bass virus (LMBV) challenge *in vivo*. Co-immunoprecipitation and immunofluorescence co-localization assays verified that MsCD40 and MsCD154 physically interact at the plasma membrane, forming a functional receptor-ligand complex. Functional assays showed that overexpression of either MsCD40 or MsCD154 significantly suppressed VHSV and LMBV infection in vitro. Furthermore, MsCD40 and MsCD154 overexpression dose-dependently activated nuclear factor-κB (NF-κB) reporter activity, and markedly upregulated the transcription of NF-κB downstream effector genes, including IL-8, NLRP3 and P105, under both VHSV and LMBV infection. Collectively, our findings demonstrate that the teleost CD40–CD154 costimulatory axis restricts both RNA and DNA viral infection in largemouth bass through NF-κB-mediated immune activation, which provides promising molecular targets for the development of broad-spectrum antiviral strategies in largemouth bass aquaculture.

## Simple Summary

Viral diseases cause devastating losses in fish aquaculture worldwide, yet effective antiviral strategies remain limited. In mammals, the CD40–CD154 protein pair serves as a central regulator of antiviral immunity, but whether this system is conserved and functional in fish was previously unknown. Using largemouth bass, a globally important farmed species, we identified CD40 and CD154, confirmed their conserved structures, and showed that both proteins are widely expressed across tissues and rapidly activated upon viral infection. We further demonstrated that CD40 and CD154 physically interact at the cell surface, and that elevating either protein significantly suppressed viral replication in fish cells. This antiviral effect operates through nuclear factor-κB, a master immune signaling regulator that switches on multiple defense genes during infection. Together, our findings reveal that the CD40–CD154 axis functions as a conserved antiviral regulatory system in largemouth bass, capable of restricting both RNA and DNA viruses.

## 1. Introduction

The Largemouth bass (*Micropterus salmoides*) is one of the most commercially important freshwater aquaculture species worldwide, with an annual production exceeding hundreds of thousands of metric tons, particularly in China, where intensive cultivation has expanded rapidly over the past two decades [1]. However, this intensification has been accompanied by an alarming increase in the incidence and severity of viral epizootics, which have become the primary limiting factor for the sustainable development of this industry [2]. Two of the most devastating pathogens for this species are viral hemorrhagic septicemia virus (VHSV, a negative-sense single-stranded RNA rhabdovirus) [3] and largemouth bass virus (LMBV, a double-stranded DNA iridovirus) [4], which cause high mortality and huge economic losses in farmed populations worldwide.

VHSV is a negative-sense single-stranded RNA virus belonging to the family *Rhabdoviridae*, genus *Novirhabdovirus*, with a broad teleost host range spanning more than 80 species across marine and freshwater fishes, including largemouth bass (*M. salmoides*), Atlantic herring (*Clupea harengus*), and rainbow trout (*Oncorhynchus mykiss*) [5]. VHSV infection causes severe hemorrhagic pathology, liver necrosis, and profound immunosuppression in susceptible fish, with mortality rates often exceeding 90% in juvenile populations [6]. LMBV is a large double-stranded DNA virus classified within the family *Iridoviridae*, genus *Ranavirus*, which is the primary etiological agent of largemouth bass disease syndrome [7]. LMBV infection causes systemic necrosis of hematopoietic and lymphoid tissues, leading to large-scale die-offs in farmed and wild populations throughout North America and Asia [8]. Notably, these two pathogens with distinct genomic characteristics and infection mechanisms simultaneously threaten largemouth bass aquaculture, creating an urgent need to identify conserved immune regulatory mechanisms that can be targeted to develop broad-spectrum antiviral strategies.

The antiviral defense system in teleost fish relies on the coordinated activation of innate and adaptive immune branches. The CD40–CD154 (CD40 ligand, CD40L) costimulatory axis is a core regulator of crosstalk between innate and adaptive immunity in vertebrates [9]. In mammals, CD40 is a type I transmembrane glycoprotein belonging to the tumor necrosis factor receptor superfamily (TNFRSF), characterized by two to four extracellular cysteine-rich pseudo-repeat domains (CRDs) that form the ligand-binding interface [10]. CD40 is constitutively expressed on antigen-presenting cells (APCs) including B cells, dendritic cells, monocytes, macrophages, as well as various non-hematopoietic cells. Upon ligand binding, CD40 recruits TNF receptor-associated factors (TRAFs) to trigger downstream signaling cascades, including Nuclear factor-κB (NF-κB), JNK/MAPK and PI3K pathways, which collectively promote APC maturation, pro-inflammatory cytokine production and cell survival [11]. CD154, the cognate ligand of CD40, is a type II transmembrane protein belonging to the TNF superfamily (TNFSF), with a conserved C-terminal TNF homology domain (THD) that forms a homotrimeric structure to engage three CD40 molecules simultaneously and initiate signal transduction [12]. CD154 is prominently expressed on activated CD4^+^ T helper cells, and the CD40–CD154 interaction mediates T cell-APC crosstalk, which is indispensable for B cell class-switch recombination, germinal center formation and protective humoral immunity against viral infection in mammals [13,14].

In teleost fish, multiple members of the TNF and TNFR superfamilies have been identified through genomic surveys and functional studies in fish species such as zebrafish (*Danio rerio*), rainbow trout (*Oncorhynchus mykiss*), Atlantic salmon (*Salmo salar*), and grass carp (*Ctenopharyngodon idella*) [15,16]. Phylogenomic analysis has revealed that TNFSF and TNFRSF repertoires expanded significantly during early teleost evolution through lineage-specific duplications, resulting in both conserved orthologues and novel paralogues with potentially divergent functions [17]. Previous studies have shown that CD40 is upregulated in mice after viral or bacterial stimulation, and its overexpression enhances interferon (IFN) production and macrophage phagocytosis [18]. In teleosts, the CD40–CD154 axis has been functionally characterized in adaptive humoral immunity in zebrafish and large yellow croaker (*Larimichthys crocea*) [10,19]. Nevertheless, specific characterization of CD40 and CD154 orthologues in perciform fish, and in particular in largemouth bass, has not been reported to date.

In the present study, we cloned and characterized the full-length coding sequences of MsCD40 and MsCD154 from largemouth bass, and analyzed their tissue distribution profiles and transcriptional dynamics in response to VHSV and LMBV challenge. We further verified the physical interaction between MsCD40 and MsCD154, and investigated their antiviral functions and downstream regulatory mechanisms. Our findings provide the functional evidence of the CD40–CD154 costimulatory axis in largemouth bass, and support its potential as a molecular target for the development of antiviral adjuvants and disease-resistant breeding strategies in largemouth bass aquaculture.

## 2. Materials and Methods

### 2.1. Viruses, Cells and Fishes

Fathead minnow (FHM) cells [20] and Largemouth bass (*M*. *salmoides*) brain (MsB) cell lines, which were derived from the brain tissue of largemouth bass, were cultured in Dulbecco’s modified Eagle’s medium (DMEM, Gibco, Waltham, MA, USA) with 15% fetal bovine serum (FBS, Gibco) at 28 °C.

VHSV (GenBank: MK598848.1) [3] and LMBV (GenBank: PX879566) [4] were propagated in FHM cells and titrated by plaque assay prior to experimental use. Polyinosinic-polycytidylic acid (poly I:C), a synthetic double-stranded RNA analog used to mimic viral nucleic acid stimulation, was purchased from Sigma-Aldrich (St. Louis, MO, USA) and dissolved in sterile phosphate-buffered saline (PBS) at a stock concentration of 1 mg/mL. All other reagents were of analytical grade unless otherwise specified.

Healthy largemouth bass (*M. salmoides*) with no visible signs of disease were obtained from a commercial aquaculture facility and maintained in recirculating freshwater tanks at 25 ± 1 °C under a 12 h light/12 h dark photoperiod. Fish were acclimated for two weeks prior to experimental use and fed a commercial pelleted diet twice daily.

### 2.2. Molecular Cloning and Sequence Analysis of MsCD40 and MsCD154

Total RNA was extracted from spleen tissue of healthy largemouth bass using TRIzol reagent (Invitrogen, Carlsbad, CA, USA) according to the manufacturer’s instructions. RNA integrity was assessed by agarose gel electrophoresis and RNA concentration was determined using a NanoDrop 2000 spectrophotometer (Thermo Fisher Scientific, Waltham, MA, USA). The full-length coding sequences of MsCD40 and MsCD154 were amplified by polymerase chain reaction (PCR) using gene-specific primers designed based on available transcriptomic data. PCR products were cloned into the pMD19-T vector (Takara Bio, San Jose, CA, USA) and confirmed by Sanger sequencing. The obtained sequences were submitted to NCBI BLAST 2.17.0 (https://blast.ncbi.nlm.nih.gov/Blast.cgi, accessed on 1 June 2026) for homology searches against the GenBank nucleotide database to verify gene identity, and multiple sequence alignments were performed using ClustalW 1.83 (https://www.genome.jp/tools-bin/clustalw, accessed on 1 June 2026). Protein domain architectures of MsCD40 and MsCD154 were predicted using the SMART v10 (http://smart.embl-heidelberg.de, accessed on 1 June 2026) and NCBI Conserved Domain Database online tools. Signal peptides and transmembrane regions were predicted using SignalP 5.0 and TMHMM 2.0, respectively.

### 2.3. Phylogenetic Analysis

The deduced amino acid sequences of MsCD40 and MsCD154 were aligned with homologous sequences retrieved from GenBank using ClustalW in MEGA 7.0 software. Phylogenetic trees were constructed using the Maximum Likelihood method with the Jones-Taylor-Thornton substitution model. Bootstrap analysis with 1000 replicates was performed to assess the statistical reliability of the tree topology. The resulting phylogenetic trees were visualized and annotated in MEGA 7.0.

### 2.4. Viral Challenge

For VHSV challenge experiments, fish were randomly divided into two groups (*n* = 30 per group): the VHSV group received intraperitoneal injection of VHSV at a dose of 1 × 10^7^ PFU per fish, and the control group received an equal volume of sterile tissue culture medium (M199). At 24 h post-injection, nine tissues were collected from both groups for gene expression analysis.

For temporal expression analysis, spleen tissues were collected from VHSV-challenged fish at 6 h, 12 h, 24 h, and 48 h post-injection (*n* = 6 per time point).

For LMBV challenge, fish were injected intraperitoneally with LMBV at a dose of 3 × 10^6^ TCID_50_ per fish in a total volume of 100 μL per fish, while the control group received an equal volume of sterile PBS buffer. The spleen tissues were collected at 6 h, 24 h, 48 h, and 72 h post-challenge (*n* = 6 per time point). To compare gene expression between healthy and diseased fish, tissues were collected from naturally infected diseased fish exhibiting clinical signs of viral disease and from age-matched healthy fish. All collected tissues were snap-frozen in liquid nitrogen and stored at −80 °C until RNA extraction.

### 2.5. Quantitative Real-Time PCR (qPCR)

Total RNA was extracted from tissues or cells using TRIzol reagent and reverse-transcribed into cDNA as described in Section 2.2. qPCR was performed on a CFX96 Real-Time PCR Detection System (Bio-Rad, Hercules, CA, USA) using TB Green Premix Ex Taq II (Takara Bio) in a total reaction volume of 20 μL. The reaction conditions were as follows: initial denaturation at 95 °C for 30 s, followed by 40 cycles of 95 °C for 5 s and 60 °C for 30 s. Primer sequences for MsCD40, MsCD154, VHSV-G, LMBV-MCP, IL-8, NLRP3, and NF-κB p105 (P105) were designed using Primer Premier 5.0 and verified for amplification efficiency (Table S1). The largemouth bass β-actin gene was used as the internal reference gene for normalization. Relative gene expression levels were calculated using the 2^−ΔΔCT^ method. Each experiment was performed with at least three independent biological replicates, and each sample was run in triplicate. Statistical significance was determined by one-way analysis of variance (ANOVA) followed by Tukey’s post hoc test, with *p* < 0.05 considered statistically significant.

### 2.6. Plasmid Construction and Transient Transfection

For overexpression studies, the full-length coding sequences of MsCD40 and MsCD154 were amplified by PCR and cloned into the pCMV-Myc or pCMV-Flag expression vectors (Clontech, Mountain View, CA, USA) to generate pCMV-Myc-MsCD154, pCMV-Myc-MsCD40, pCMV-Flag-MsCD40, and pCMV-Flag-MsCD154 constructs, respectively. Empty pCMV-Myc and pCMV-Flag vectors were used as negative controls. All constructs were verified by restriction enzyme digestion and Sanger sequencing prior to use.

Transient transfection of fish cell lines was performed using Lipofectamine 3000 (Invitrogen) according to the manufacturer’s protocol. Cells were seeded in 6-well or 24-well plates at 70–80% confluence and transfected with the indicated constructs at a DNA concentration of 1–2 μg per well. Cells were harvested at 24–48 h post-transfection for downstream analyses.

### 2.7. Dual-Luciferase Reporter Assay

To assess the effects of MsCD40 and MsCD154 on NF-κB transcriptional activity, cells were co-transfected with an NF-κB-responsive firefly luciferase reporter plasmid (pNF-κB-Luc), a Renilla luciferase plasmid (pRL-TK, Promega, Madison, WI, USA) as an internal transfection control, and increasing doses of pCMV-Myc-MsCD40 or pCMV-Myc-MsCD154 expression constructs. At 24 h post-transfection, cells were infected with VHSV or LMBV at the indicated multiplicity of infection (MOI). At the designated time points post-infection, cells were lysed using passive lysis buffer, and luciferase activities were measured using the Dual-Luciferase Reporter Assay System (Promega) on a GloMax 20/20 luminometer (Promega). Firefly luciferase activity was normalized to Renilla luciferase activity to correct for transfection efficiency, and NF-κB relative luciferase activity was calculated relative to the empty vector control group. Each experiment was performed in triplicate and repeated at least three times independently.

### 2.8. Western Blot Analysis

Cells or tissues were lysed in RIPA lysis buffer (Beyotime, Shanghai, China) supplemented with protease inhibitor cocktail (Roche, Basel, Switzerland) on ice for 30 min, followed by centrifugation at 12,000× *g* at 4 °C for 15 min. Protein concentrations were determined by the BCA protein assay kit (Thermo Fisher Scientific). Equal amounts of protein (30–50 μg per lane) were separated by 10–12% SDS-PAGE and transferred onto polyvinylidene fluoride (PVDF) membranes (Millipore, Burlington, MA, USA). Membranes were blocked with 5% non-fat dry milk in Tris-buffered saline containing 0.1% Tween-20 (TBST) at room temperature for 1 h, followed by overnight incubation at 4 °C with the following primary antibodies: anti-Myc (1:1000; Cell Signaling Technology, Danvers, MA, USA), anti-Flag (1:1000; Sigma-Aldrich), anti-VHSV-G (1:500; prepared as described), and anti-β-actin (1:5000; Abcam, Cambridge, UK). After three washes with TBST, membranes were incubated with horseradish peroxidase (HRP)-conjugated secondary antibodies (1:5000) at room temperature for 1 h. Protein bands were visualized using an enhanced chemiluminescence (ECL) detection system (Thermo Fisher Scientific) and imaged with a ChemiDoc XRS+ system (Bio-Rad). Band intensities were quantified using ImageJ software v1.54t (NIH, Bethesda, MD, USA) and normalized to β-actin as the loading control.

### 2.9. Immunofluorescence Microscopy

To determine the subcellular localization of MsCD40 and MsCD154, cells were seeded on glass coverslips in 24-well plates and co-transfected with pCMV-Flag-MsCD40 and pCMV-Myc-MsCD154 plasmids. At 24 h post-transfection, cells were processed under two conditions: (1) permeabilization with 0.1% Triton X-100 in PBS for 10 min at room temperature to allow intracellular antibody access, and (2) non-permeabilization to restrict antibody binding to cell surface proteins. In both conditions, cells were fixed with 4% paraformaldehyde in PBS for 15 min, washed three times with PBS, and blocked with 5% bovine serum albumin in PBS for 1 h at room temperature. Cells were then incubated overnight at 4 °C with anti-Flag antibodies (1:500; Sigma-Aldrich) to detect MsCD40 and anti-Myc antibodies (1:500; Cell Signaling Technology) to detect MsCD154. After washing, cells were incubated with Alexa Fluor 555-conjugated anti-mouse IgG (red, 1:1000; Invitrogen) and Alexa Fluor 488-conjugated anti-rabbit IgG (green, 1:1000; Invitrogen) secondary antibodies for 1 h at room temperature in the dark. Nuclei were counterstained with DAPI (1 μg/mL) for 10 min. Coverslips were imaged using a confocal laser scanning microscope (Leica SP8, Nussloch, Germany). Co-localization was assessed by visual inspection of merged fluorescence channels.

### 2.10. Co-Immunoprecipitation (Co-IP)

To verify the physical interaction between MsCD40 and MsCD154, Co-IP experiments were performed using cells co-transfected with pCMV-Flag-MsCD40 and pCMV-Myc-MsCD154, or the respective single-construct and empty vector controls as previously described [21]. At 48 h post-transfection, cells were lysed in Co-IP lysis buffer (25 mM Tris-HCl pH 7.4, 150 mM NaCl, 1% NP-40, 1 mM EDTA, 5% glycerol) supplemented with protease inhibitor cocktail on ice for 30 min, followed by centrifugation at 12,000× *g* at 4 °C for 15 min. For immunoprecipitation, cell lysates were incubated with anti-Flag/Myc magnetic beads (MedChemExpress, Monmouth Junction, NJ, USA) for 16 h at 4 °C with rotation. Beads were washed five times with lysis buffer, and immunoprecipitated proteins were eluted by boiling in 2 × SDS loading buffer for 10 min. Co-precipitated proteins were analyzed by Western blot as described in Section 2.10, using anti-Myc and anti-Flag antibodies to detect co-immunoprecipitated MsCD154 and MsCD40 proteins, respectively. Total cell lysates (Input) were included as positive controls to confirm protein expression.

### 2.11. Statistical Analysis

All quantitative data are presented as mean ± standard error of the mean (SEM) from at least three independent biological replicates. Technical replicates were performed within each biological replicate to ensure measurement precision. Statistical comparisons between two groups were performed using the unpaired Student’s *t*-test, and comparisons among multiple groups were conducted using one-way ANOVA followed by Tukey’s honestly significant difference post hoc test. Prior to parametric testing, normality of all datasets was confirmed using the Shapiro–Wilk test (*p* > 0.05), and homogeneity of variance across groups was verified using Levene’s test (*p* > 0.05). For time-course infection experiments, two-way ANOVA, followed by Tukey’s post hoc test was used for multiple comparisons. All statistical analyses were performed using GraphPad Prism 8.0. A *p*-value of less than 0.05 was considered statistically significant. Significance levels are indicated as * *p* < 0.05 and ** *p* < 0.01.

## 3. Results

### 3.1. Molecular Characterization and Phylogenetic Analysis of MsCD40 and MsCD154

**Figure 1:**
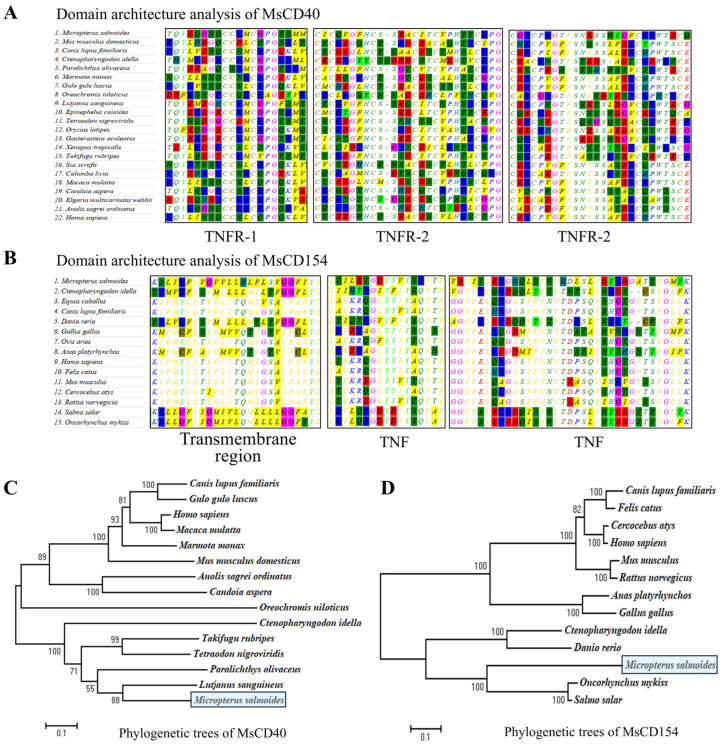
Molecular characterization and phylogenetic analysis of CD40 and CD154 in largemouth bass (*Micropterus salmoides*). (**A**,**B**) Domain architecture analysis of MsCD40 and MsCD154 domains against known sequences in the NCBI database. MsCD40 harbors canonical tumor necrosis factor receptor (TNFR) cysteine-rich domains; MsCD154 contains conserved TNF homology domains and a transmembrane region. (**C**,**D**) Maximum likelihood phylogenetic trees of MsCD40 and MsCD154; numbers at nodes indicate bootstrap support values; largemouth bass (*M. salmoides*) sequences are marked with solid squares.

The open reading frames of *MsCD40* and *MsCD154* were 725 bp and 998 bp in length, encoding 241 and 332 amino acids, respectively. Domain architecture analysis revealed that MsCD40 harbors multiple cysteine-rich TNFR domains and a transmembrane region, consistent with the canonical architecture of TNF receptor superfamily members (Figure 1A). MsCD154 contains a C-terminal TNF homology domain and a transmembrane region, characteristic of TNF superfamily ligands (Figure 1B).

Phylogenetic analysis was performed to investigate the evolutionary relationship of MsCD40 and MsCD154 with their homologs from other vertebrates. The results showed that MsCD40 clustered with teleost CD40 homologs, with the closest relationship to those from red snapper (*Lutjanus sanguineus*) and Japanese flounder (*Paralichthys olivaceus*) (Figure 1C). MsCD154 grouped with teleost CD154 homologs from salmonids, zebrafish and grass carp, and formed a well-supported teleost-specific clade that was clearly distinct from mammalian and avian counterparts (Figure 1D). These results demonstrate that MsCD40 and MsCD154 possess structurally conserved domain architectures and represent evolutionarily conserved members of their respective protein superfamilies within teleosts.

### 3.2. Expression Profiles of MsCD40 and MsCD154 in M. salmoides and in Response to Viral Challenge

**Figure 2:**
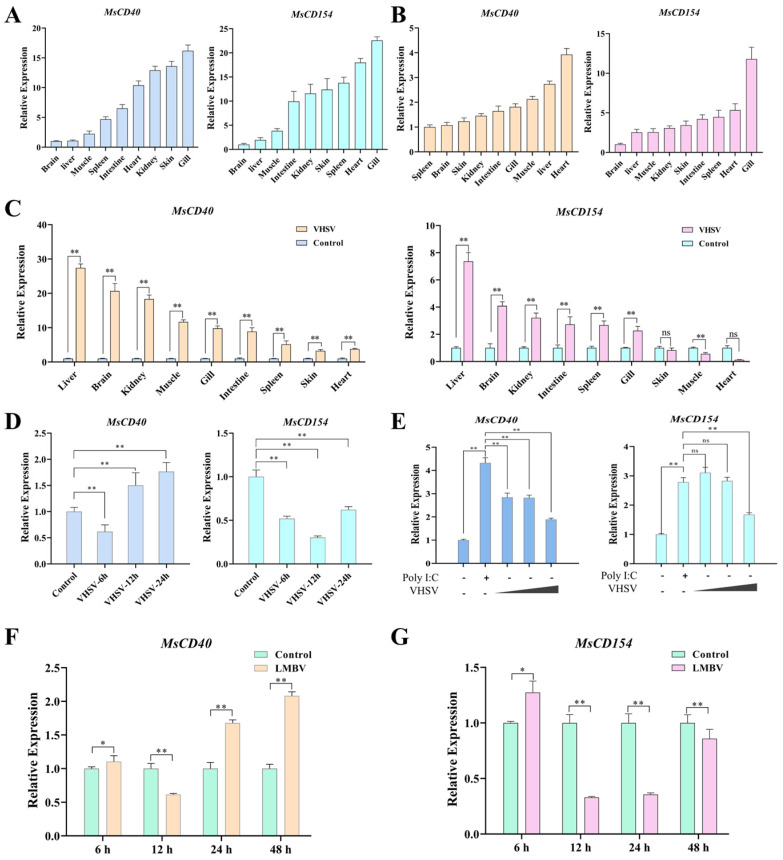
Tissue expression profiles of *MsCD40* and *MsCD154* in largemouth bass and transcriptional responses to viral stimulation. (**A**,**B**) Relative expression of *MsCD40* and *MsCD154* across nine tissues in healthy fish and at 24 h following intraperitoneal injection of VHSV. (**C**) Comparative expression of *MsCD40* and *MsCD154* between healthy and diseased fish (*n* = 30) across multiple tissues. (**D**) Temporal expression dynamics of *MsCD40* and *MsCD154* in spleen at 6 h, 12 h, 24 h, and 48 h post-VHSV challenge (*n* = 30). (**E**) Expression changes of *MsCD40* and *MsCD154* in MsB cells treated with poly I:C or VHSV at varying doses. The triangle represents increasing multiplicities of infection (MOI) of VHSV, corresponding to MOI = 2, 5, and 7. (**F**,**G**) Relative expression levels of *MsCD40* and *MsCD154* in spleen at 6 h, 12 h, 24 h, and 48 h post-LMBV challenge; PBS-injected fish served as the control group. ns, no significant difference, * *p* < 0.05, ** *p* < 0.01; data are presented as mean ± SEM of three independent biological replicates (*n* = 3).

To explore the potential immune function of MsCD40 and MsCD154, we first detected their basal expression levels in nine tissues of healthy largemouth bass. Both *MsCD40* and *MsCD154* were ubiquitously expressed in all detected tissues, with the highest expression levels in immune-relevant tissues including gill, heart, head kidney, spleen and liver (Figure 2A). We further investigated the transcriptional responses of *MsCD40* and *MsCD154* to VHSV and LMBV challenge in vivo. Following VHSV infection, the expression of *MsCD40* and *MsCD154* was significantly upregulated in gill, heart and liver at 24 h post-infection (hpi), compared with the control group (Figure 2B,C). Temporal expression analysis in spleen showed that *MsCD40* expression was slightly suppressed at 6 hpi, and then significantly upregulated with a peak at 24 hpi, followed by a decline at 48 hpi (Figure 2D). *MsCD154* expression was transiently downregulated at 6 hpi, and then gradually recovered to the basal level at 48 hpi (Figure 2D). In addition, VHSV infection activated the transcription of *MsCD40* and *MsCD154* in MsB cells, similar to poly I:C stimulation, but led to a decrease in protein expression as the viral dose increased (Figure 2E).

Following LMBV challenge, *MsCD40* exhibited a biphasic expression pattern in spleen: it was slightly upregulated at 6 hpi, downregulated at 12 hpi, and then significantly increased at 24 hpi and 48 hpi (Figure 2F). *MsCD154* expression was transiently upregulated at 6 hpi, significantly suppressed at 12 hpi and 24 hpi, and partially recovered at 48 hpi (Figure 2G). Taken together, these results indicate that *MsCD40* and *MsCD154* are widely expressed in immune-relevant tissues and dynamically regulated by viral infection, suggesting their potential involvement in the antiviral immune response of largemouth bass.

### 3.3. MsCD40 and MsCD154 Exert Broad-Spectrum Antiviral Activity Against VHSV and LMBV

**Figure 3:**
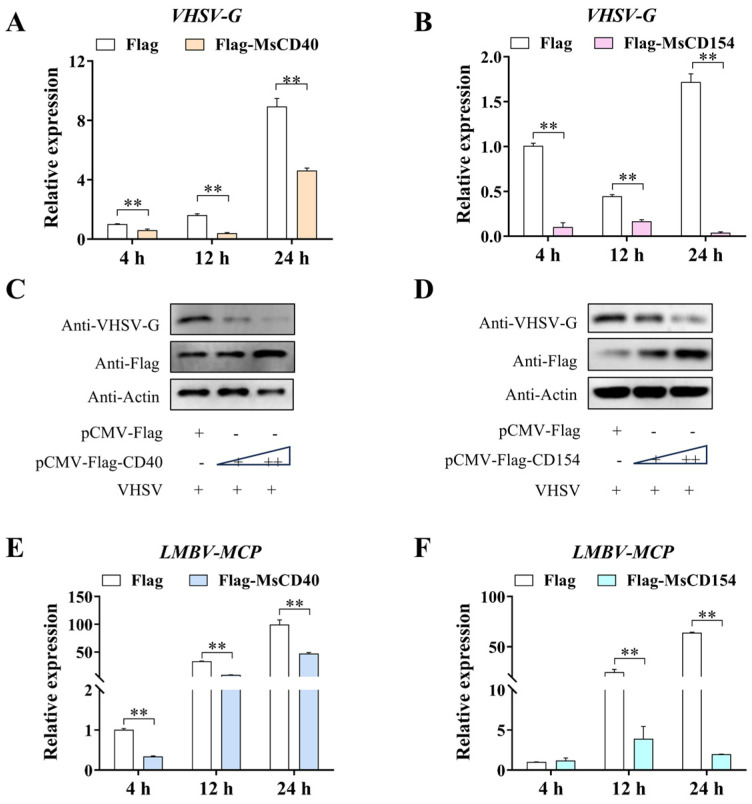
The effects of MsCD40 and MsCD154 on VHSV and LMBV challenge. (**A**,**B**) *VHSV-G* mRNA levels (qPCR) in MsB cells overexpressing MsCD40 or MsCD154 at 4 h, 12 h, and 24 h post-VHSV infection, ** *p* < 0.01; data are presented as mean ± SEM. (**C**,**D**) The protein levels (WB) in FHM cells overexpressing MsCD40 or MsCD154 (250 or 500 ng) at 24 h post-VHSV infection, bands include Anti-VHSV-G, Anti-Flag, and Anti-Actin. The triangle represents increasing plasmid transfection doses, corresponding to 250 ng and 500 ng. (**E**,**F**) Relative expression of LMBV major capsid protein (*MCP*) in MsB cells overexpressing MsCD40 (Flag-MsCD40) or MsCD154 (Flag-MsCD154) at 4 h, 12 h, and 24 h post-infection; white bars represent the empty vector control (Flag). ** *p* < 0.01; data are presented as mean ± SEM.

To verify the antiviral function of MsCD40 and MsCD154, we overexpressed MsCD40 or MsCD154 in MsB cells, and then infected the cells with VHSV or LMBV. The results showed that overexpression of either MsCD40 or MsCD154 significantly suppressed the mRNA level of *VHSV-G* at 4, 12 and 24 hpi, compared with the empty vector control group (Figure 3A,B). Western blot analysis further confirmed that the protein abundance of VHSV-G was markedly reduced in MsCD40 and MsCD154 overexpression groups in a dose-dependent manner (Figure 3C,D).

Similarly, overexpression of MsCD40 or MsCD154 significantly inhibited the transcription of *LMBV-MCP* at 4, 12 and 24 hpi, and the inhibitory effect increased progressively over time (Figure 3E,F). These results demonstrate that both MsCD40 and MsCD154 exert significant antiviral activity against both VHSV (RNA virus) and LMBV (DNA virus) in vitro.

### 3.4. MsCD40 Physically Interacts with MsCD154 at the Plasma Membrane

**Figure 4:**
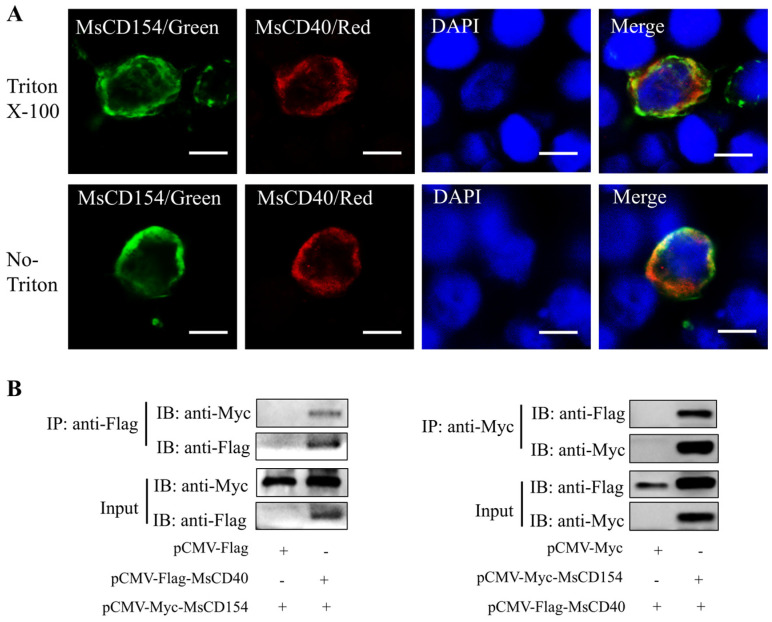
Subcellular localization and protein interaction of MsCD40 and MsCD154. (**A**) Immunofluorescence analysis of MsCD40 and MsCD154 localization under Triton X-100 permeabilization and non-permeabilization (No Triton) conditions; MsCD154 was detected with anti-Myc antibody (green), MsCD40 with anti-Flag antibody (red), and nuclei were stained with DAPI (blue); Merge panels show channel overlay. Scale bar = 8 μm. (**B**) Co-immunoprecipitation (Co-IP) combined with Western blot to verify the physical interaction between MsCD40 and MsCD154. Immunoprecipitations were detected with anti-Flag or anti-Myc antibodies.

To form a functional signaling axis, CD40 and CD154 must interact at the cell surface as a receptor-ligand complex. We thus performed immunofluorescence co-localization and Co-IP assays to verify the interaction between MsCD40 and MsCD154. Immunofluorescence analysis showed that MsCD40 (red) and MsCD154 (green) were predominantly co-localized at the cell periphery and plasma membrane under both permeabilized and non-permeabilized conditions, with obvious yellow signals in the merged images (Figure 4A).

Reciprocal Co-IP assays further confirmed the physical interaction between MsCD40 and MsCD154: anti-Flag immunoprecipitation of Flag-MsCD40 successfully co-precipitated Myc-MsCD154, and anti-Myc immunoprecipitation of Myc-MsCD154 reciprocally co-precipitated Flag-MsCD40, while no specific co-precipitation signal was detected in the negative control groups (Figure 4B). These results demonstrate that MsCD40 and MsCD154 can bind to each other and form a functional receptor-ligand complex at the plasma membrane.

### 3.5. MsCD40 and MsCD154 Activate the NF-κB Signaling Pathway

**Figure 5:**
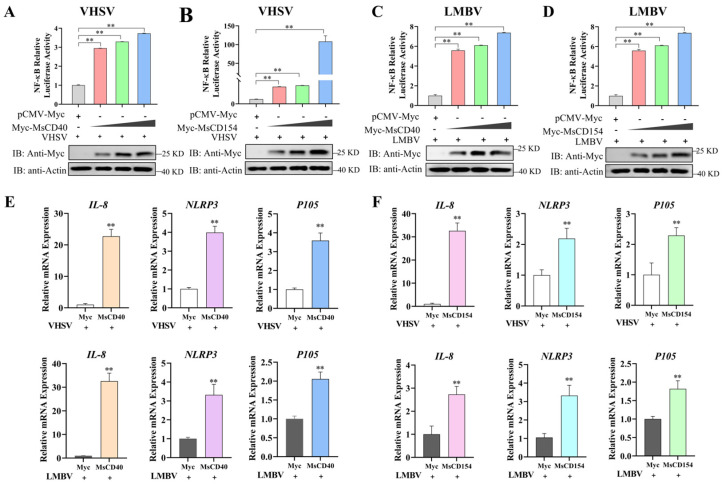
Effects of MsCD40 and MsCD154 on NF-κB signaling pathway activity. (**A**,**B**) Dual-luciferase reporter assays measuring NF-κB promoter activity following dose-gradient overexpression of MsCD40 (Myc-tagged) (**A**) or MsCD154 (Myc-tagged) (**B**) under VHSV infection conditions. (**C**,**D**) NF-κB promoter activity following overexpression of MsCD40 (Myc-tagged) (**C**) or MsCD154 (Myc-tagged) (**D**) under LMBV infection conditions. Western blot panels below each luciferase graph (Anti-Myc, Anti-Actin) confirm recombinant protein expression and equal loading. The triangle represents increasing plasmid transfection doses, corresponding to 100 ng 200 ng, and 300 ng. (**E**,**F**) qPCR analysis of NF-κB downstream target genes *IL-8*, *NLRP3*, and *P105* mRNA levels in cells overexpressing MsCD40 (**E**) or MsCD154 (**F**) under VHSV and LMBV infection conditions. ** *p* < 0.01; data are presented as mean ± SEM.

The NF-κB signaling pathway is the primary downstream cascade of the CD40–CD154 axis in mammals, which mediates the transcription of immune effector genes. We thus investigated whether MsCD40 and MsCD154 activate NF-κB signaling in largemouth bass during viral infection. Dual-luciferase reporter assays showed that overexpression of MsCD40 or MsCD154 dose-dependently enhanced NF-κB reporter activity by 3- to 120-fold under VHSV infection, compared with the control group (Figure 5A,B). Consistent results were observed under LMBV infection, where overexpression of MsCD40 or MsCD154 also significantly elevated NF-κB promoter activity in a dose-dependent manner (Figure 5C,D). The expression of recombinant MsCD40 and MsCD154 proteins was confirmed by Western blot (Figure 5A–D).

Furthermore, qPCR analysis showed that overexpression of MsCD40 or MsCD154 significantly upregulated the mRNA levels of well-characterized NF-κB target genes, including *IL-8*, *NLRP3* and *P105*, under both VHSV and LMBV infection conditions (Figure 5E,F). Collectively, these results demonstrate that MsCD40 and MsCD154 function as positive regulators of the NF-κB signaling pathway, and activate the transcription of downstream antiviral immune effector genes in largemouth bass.

## 4. Discussion

The CD40–CD154 costimulatory axis is a pivotal regulator of innate and adaptive immunity in vertebrates, but its functional role in teleost antiviral immunity, especially in perciform fish, remains poorly characterized. In this study, we cloned and functionally characterized MsCD40 and MsCD154 from largemouth bass, a commercially important freshwater species highly susceptible to both VHSV and LMBV. Our results demonstrate for the first time that the CD40–CD154 axis is functionally conserved in largemouth bass, which confers broad-spectrum antiviral activity against both RNA and DNA viruses via activating the NF-κB signaling pathway. These findings advance our understanding of teleost costimulatory immune regulation, and provide promising molecular targets for antiviral strategy development in largemouth bass aquaculture.

Bioinformatic and phylogenetic analysis confirmed that MsCD40 and MsCD154 possess the canonical domain architectures of TNFRSF and TNFSF members, respectively, and clustered with other teleost homologs in a lineage-specific clade. The conserved CRDs in MsCD40 and THD in MsCD154 are essential for the receptor-ligand interaction and subsequent signal transduction, which is consistent with the structural characteristics reported in mammals and other teleost species [22,23]. The teleost-specific phylogenetic clustering pattern reflects the evolutionary divergence of CD40 and CD154 between teleost and tetrapod, which is consistent with previous findings that TNFSF/TNFRSF members underwent lineage-specific duplications during early teleost evolution [24]. This evolutionary divergence also underscores the necessity of direct functional characterization in largemouth bass, rather than extrapolating functions from mammalian or other teleost models.

The broad tissue distribution of MsCD40 and MsCD154 in healthy largemouth bass, with enriched expression in gill, kidney, spleen, and heart, is consistent with the known distribution of CD40 and CD154 in immune-competent and barrier tissues of other vertebrates [25]. Notably, the high expression of MsCD40 and MsCD154 in gill and skin, which are the primary mucosal barriers against aquatic pathogens, indicates that this axis may play an important role in mucosal antiviral immunity of largemouth bass. The comparatively lower expression in brain and liver under homeostatic conditions mirrors observations in mammals, where CD40 expression is restricted at immunologically privileged sites [26,27]. Following VHSV and LMBV challenge, MsCD40 and MsCD154 exhibited dynamic biphasic expression patterns, with early transient suppression followed by significant upregulation at later infection stages. This expression pattern is consistent with the model of initial viral-mediated immune evasion and subsequent host compensatory immune activation, which has also been observed for other TNFSF/TNFRSF members during viral infection in teleost [28,29]. The divergence in temporal kinetics between MsCD40 and MsCD154 further suggests that the two molecules are governed by distinct transcriptional programs during the antiviral response, potentially involving differential engagement of upstream cascades such as IRF3/7 or NF-κB at specific infection stages [30]. The responsiveness of both molecules to poly I:C stimulation confirms that their regulation is intrinsic to immune cells rather than a secondary consequence of systemic physiological perturbation.

A prerequisite for the functional CD40–CD154 axis is the physical interaction between the receptor and ligand at the cell surface. Our immunofluorescence and Co-IP results confirmed that MsCD40 and MsCD154 co-localized at the plasma membrane and bound to each other, which is consistent with the interaction pattern reported in mammals and zebrafish [9,10], indicating that the receptor-ligand interaction mode of the CD40–CD154 axis is evolutionarily conserved from teleost to mammals. The conservation of this membrane-level interaction across vertebrate lineages, including amphibians and cartilaginous fish where CD40–CD154 homologs have also been identified, suggests that this costimulatory axis emerged early in vertebrate evolution and was diversified to meet the immunological demands of distinct ecological niches [30]. Future structural studies, particularly cryo-electron microscopy of the MsCD40–MsCD154 complex, will be essential to delineate the precise molecular interface governing this interaction.

Functional assays showed that overexpression of MsCD40 or MsCD154 significantly suppressed the replication of both VHSV and LMBV in vitro, which is the first direct evidence that the CD40–CD154 axis exerts antiviral activity against both RNA and DNA viruses in teleost. Previous studies in mice have shown that CD40 signaling restricts RNA virus replication in macrophages by inducing rapid innate immune activation [18], and our findings extend this antiviral function to teleost fish. The broad-spectrum antiviral activity of the CD40–CD154 axis makes it a promising target for the development of universal antiviral strategies against the two most devastating viral pathogens in largemouth bass aquaculture.

To further elucidate the molecular mechanism underlying the antiviral activity of the CD40–CD154 axis, we investigated its regulatory effect on the NF-κB signaling pathway, which is the core downstream cascade of CD40–CD154 in mammals. In mammals, CD40–CD154 engagement activates NF-κB through TRAF2/3/5/6-mediated signaling, driving transcription of pro-inflammatory and pro-survival genes [31,32]. Our results showed that overexpression of MsCD40 or MsCD154 dose-dependently activated NF-κB reporter activity, and upregulated the transcription of NF-κB downstream target genes IL-8, NLRP3 and P105 under both VHSV and LMBV infection. These findings indicate that the activation of NF-κB signaling is conserved as the core downstream mechanism of the CD40–CD154 axis in teleosts. IL-8 is a key chemokine for neutrophil recruitment and inflammatory response initiation [33], NLRP3 is a core component of the inflammasome that mediates antiviral innate immunity [34], and P105 is the precursor of NF-κB p50 subunit that regulates NF-κB autoregulation [35]. The upregulation of these genes suggests that the CD40–CD154 axis activates a broad inflammatory and antiviral immune response via NF-κB signaling, thereby restricting viral replication. And it is important to note that overexpression approaches drive target gene expression at levels substantially exceeding those achieved under endogenous regulation, as transgene transcription is governed by strong exogenous promoters rather than native regulatory elements. Therefore, the overexpression data presented in this study should be interpreted as an initial functional demonstration of the antiviral potential of MsCD40 and MsCD154, rather than a complete recapitulation of their endogenous regulatory roles. To substantiate the physiological relevance of these findings, future studies employing loss-of-function approaches, including CRISPR/Cas9-mediated gene knockout or RNA interference-based knockdown, will be essential to evaluate the contribution of MsCD40 and MsCD154 to antiviral immunity under conditions that more faithfully reflect their native expression context.

## 5. Conclusions

In summary, this study demonstrates that the CD40–CD154 costimulatory axis is functionally conserved in largemouth bass, which physically interacts at the plasma membrane, activates NF-κB signaling and downstream antiviral immune response, and confers broad-spectrum antiviral activity against both VHSV and LMBV. These findings advance our understanding of teleost costimulatory immune regulation, and provide a molecular foundation for the development of broad-spectrum antiviral strategies in largemouth bass aquaculture.

## Data Availability

The original contributions presented in this study are included in the article/Supplementary Material. Further inquiries can be directed to the corresponding authors.

## Supplementary Materials

The following supporting information can be downloaded at: https://www.mdpi.com/article/10.3390/ani16111719/s1, Table S1: Primer sequences used in this study.

## Abbreviations

The following abbreviations are used in this manuscript:

VHSV	Viral hemorrhagic septicemia virus
LMBV	Largemouth bass virus
NF-κB	Nuclear factor-κB
TNFRSF	Tumor necrosis factor receptor superfamily
CRDs	Extracellular cysteine-rich pseudo-repeat domains
APCs	Antigen-presenting cells
TRAFs	TNF receptor-associated factors
TNFSF	TNF superfamily
THD	TNF homology domain
IFN	Interferon
FHM	Fathead minnow cell
MsB	Largemouth bass (*Micropterus salmoides*) brain
DMEM	Dulbecco’s modified Eagle’s medium
FBS	Fetal bovine serum
poly I:C	Polyinosinic-polycytidylic acid
PBS	Phosphate-buffered saline
PCR	Polymerase chain reaction
PVDF	Polyvinylidene fluoride
SEM	Standard error of the mean
